# Perspectives on Inequity and Health Disparities in Chile and Their Relationship to Microbial Ecology

**DOI:** 10.1128/msystems.01496-21

**Published:** 2022-09-29

**Authors:** José Izcue, Ismael Palacios-García, Felipe Rojas Traverso, Macarena Koller, Francisco J. Parada

**Affiliations:** a Departamento de Medicina Familiar, Universidad de los Andes, Santiago, Chile; b Escuela de Psicología, Pontificia Universidad Católica de Chile, Santiago, Chile; c Centro de Estudios en Neurociencia Humana y Neuropsicología, Facultad de Psicología, Universidad Diego Portales, Santiago, Chile; d Escuela de Medicina, Universidad de los Andes, Santiago, Chile; University of Maine

**Keywords:** health, microbiota, inequity, Chile, green space, lifestyle

## Abstract

Among countries in the Organisation for Economic Cooperation and Development (OECD), Chile stands out as having important inequalities in income distribution, dietary quality, access to urban green spaces, and health outcomes. People in lower socioeconomic groups consistently show higher rates of noncommunicable chronic diseases and are being hit the hardest by the COVID-19 pandemic. These chronic conditions are increasingly considered to be shaped, or affected by, the human gut microbiome. Moreover, inequity as an overarching concept might also be associated with microbial patterns and if so, this may represent a novel pathway through which to address health and other disparities. Focusing on the case of Chile, our goal is to contribute to a critical discussion and motivate researchers and policymakers to consider the role of the microbiome in social equity in future endeavors.

## PERSPECTIVE

Noncommunicable chronic diseases (NCDs) are a major contributor to the burden of disease worldwide (1). Despite their widespread prevalence, the distribution of these conditions is not equitable across countries and differs greatly within populations according to differences in socioeconomic status (SES) and other social determinants (2). A relative newcomer in the understanding of the mechanisms underlying NCD development is the gut microbiome, and differences in the prevalence of these diseases across communities appear to be influenced by it (3). The relationship between health and the human gut microbiome has recently received increasing attention (4). It is now widely acknowledged that several factors influence its composition, including delivery method at birth, breastfeeding, exposure to antibiotics during infancy, nutrition, and urbanization/exposure to green spaces, among others (5).

In this paper, we discuss to what extent inequity and the microbiome are linked as risk factors for health status, with a distinct focus on Chile. First, we review health and other inequities within the Chilean population. We then provide an overview of global human microbiome determinants. Finally, we explore potential pathways linking these inequities and the microbiome in the Chilean context.

## INEQUITY IN CHILE

Among the Organisation for Economic Co-operation and Development (OECD), Chile is one of the most unequal countries (6). It is estimated that the richest 1% captures 17% of the total tax revenue, while the wealthiest 10% receives over 50% of all income (7). The origins of inequality in Chile can be traced back to European colonialism, the consequences of which are still prominent today (8). Notable disparities encompass culture, health care access, nutrition, air pollution, and access to green spaces, among others (9). Inequities within these dimensions may have an impact on Chileans’ microbiomes and will now be reviewed.

### Inequality of green spaces.

According to the World Health Organization (WHO), every city is recommended to provide a minimum of 9 m^2^ of urban green space per capita (10, 11). Chile’s Metropolitan region (where the capital Santiago is located) has an average of 3.2 m^2^ of urban green space per capita, with a very unequal distribution (12). One study showed the poorest sectors’ ratio ranged between 0.4 and 2.9 m^2^/person, while higher-income areas’ ratio was 6.7 to 18.8 m^2^/person (13). Distance to green space from a given household is larger in poorer sectors and accessibility is worse (13). Additionally, low-income neighborhoods have much lower vegetation or green cover in their parks or public squares, compared with higher-income areas. Vegetation cover ranges from less than 27% in poorer districts to over 70% in the wealthiest (14). Plant species’ richness yields greater soil microbial diversity, which has potential implications for human health and health equity (15). It follows that this disparity may have tangible health benefits for the rich, and detriments for the poor.

### Health disparities.

Chile has a high prevalence of various NCDs. The prevalence of obesity is 34.4% while 39.8% of the population is overweight (16), representing the second-highest excess weight prevalence within OECD countries (17). The prevalence of type 2 diabetes in 2017 was 12.3%, rising from 9.4% in 2010 (18). This places Chile as the sixth country with the highest diabetes rates among the OECD (19).

In the realm of mental health, depressive disorders are some of the most widespread diseases, showing a prevalence of 6,8%, according to the National Health Survey from 2017 (16). Data disaggregated by gender reveals a much higher prevalence of depression among Chilean women compared with Chilean men: 10.1% versus 2.1%, respectively. These diseases exist within a *social gradient* (20), where a lower income and overall SES correlate with a higher prevalence of NCDs, while the opposite is also true. Unsurprisingly, lifestyle-related risk factors for these chronic diseases, such as low levels of physical activity and poor dietary quality, are more widespread in Chileans of lower SES (21, 22).

Obesity, diabetes, other NCDs, and depression are to some extent mediated by the gut microbiome. The behavioral and environmental risk factors that catalyze the onset of these diseases have an impact on the gut microbiome (23). The next section will consider some of these microbiome determinants and then discuss potential links to health inequities in Chile.

## MICROBIOME DETERMINANTS ACROSS THE LIFE-COURSE

### First microbiome determinants.

Childbirth delivery method is considered the first major microbiome determinant (24). While natural delivery allows the vertical maternal-newborn transmission of vaginal microbiota, caesarean delivery is characterized by colonization of skin microorganisms (24), which may have implications for asthma, allergies, and other inflammatory conditions (25). Breastfeeding is considered a protective factor for those conditions and represents the main microbiota covariate through the first year of life (26), regardless of delivery method. This might have important health implications in Chile, where breastfeeding rates at the sixth month of life are estimated at 44% (27, 28). Other microbiome determinants such as antibiotic use during childhood may contribute to conditions such as obesity and becoming overweight (29), but data on this relationship is scarce in Chile.

### Nutrition and the gut microbiome.

During adolescence, the microbiome is influenced by several physiological and environmental cues, which slowly change and develop into adulthood (30). In adulthood, an important determinant of the gut environment is diet (31). Eating patterns high in whole grains, fruits, and vegetables, such as the Mediterranean diet, have been associated with increased microbial diversity and stability (32). Conversely, western diets (i.e., high in ultraprocessed foods) or fad diets (e.g., ketogenic, paleolithic) are related to diminished microbial abundance and diversity (33). Diets lacking enough fiber content decrease the gut’s mucus thickness and induce a “leaky gut” (34) and the expression of proinflammatory markers (35). Conversely, fiber intake favors “beneficial” bacteria growth, particularly those species specialized in short-chain fatty acid production such as butyrate, acetate, and propionate (36). Cross-sectional studies in Chile show that among preschool children, almost 50% of dietary calories come from ultraprocessed foods low in fiber (37). In adults, this amounts to almost 30% of total calorie intake (38, 39).

### Urbanization and green space exposure.

The built environment exerts an important influence on the human microbiome across the life-course, as both host and environmental microbiomes are interconnected and exchange microorganisms regularly (40). Urban sectors often have depleted microbial biodiversity (41–43), which is relevant because this influences the composition of the human gut, nasal, and skin microbiomes, and because a diverse gut microbiome has been identified as a health predictor (44). Furthermore, studies looking at the effects of the aerobiome (the collection of microorganisms in a given airspace) on murine models have shown that it affects their guts’ microbial composition, short-chain fatty acid production, and has anxiolytic effects (45). Also, evidence shows vertical stratification of the vegetation in greenspaces influences the aerobiome composition, with potential implications for human health (46).

Over half of the global population lives in urban areas and over two-thirds are predicted to embrace urbanization by midcentury (https://ourworldindata.org/grapher/urban-and-rural-population-2050?country=~OWID_WRL). Chile is no exception, as 88.6% of its population lives in urban areas and this number is projected to continue rising (47). Urbanization disturbs soil properties and alters the diversity of its microbial communities (48). The loss of microbial diversity has potential consequences for urban dwellers’ microbiomes and it has been called a “*major public health threat”* by researchers (49).

Despite the influence of these microbiome determinants, pharmaceuticals appear to be stronger drivers of the composition of the gut microbiome in adults. This has been the case in Western European and American cohorts (50, 51). Nevertheless, the role of lifestyle and environmental exposures should not be underestimated, as studies looking at the gut microbiome of hunter-gatherers’ communities show important differences with urban controls (43). Interventions modifying the biodiversity of the built environment show that this has an impact on the hosts’ skin and gut microbiomes (52).

Contrasting social realities are underpinned by different lifestyles and environmental exposures. Potential links between the microbiome and health and social inequities will now be explored.

## IS THERE A RELATIONSHIP BETWEEN INEQUITY AND THE HUMAN MICROBIOME?

To date, most of the studies on the human microbiome have been conducted in European (53) or North American populations (54). Few studies look at the microbiomes of Latin Americans or specifically Chileans (55). This issue is likely worse for ethnic minorities in Chile and even more so among indigenous communities. Learning more about the microbiota of Chilean individuals and communities is key to better understanding the potential relationships between health, inequity, and local gut microbiomes.

Lower-income citizens live in areas lacking enough urban green space and with less diverse vegetation. Close contact with nature has been shown to reduce the risk of mental illnesses (e.g., depressive disorder) and it is also a protective factor against NCDs (e.g., hypertension, cardiovascular risk factors) (56, 57). This happens through various pathways, some of which involve interactions between host and environmental microbiomes (58, 59). Therefore, inequities in green space distribution and access are likely to promote a less biodiverse gut microbiome among the most disadvantaged, with potentially negative consequences for their health (58).

On the other hand, Chileans of low SES consume unhealthy diets low in fiber and high in processed foods (16), which are an established risk factor for several NCDs (60, 61). One of the underlying physiological mechanisms leading from unhealthy diets to chronic diseases is the microbiome, as a lack of dietary fiber and the intake of processed foods’ ingredients have been shown to have a negative impact on the microbiomes of both animal and human models (62–64). These data and relationships raise a very important possibility; the *social gradient*, whereby lower-income populations in Chile have a higher prevalence of NCDs with a lower life expectancy (65), might also exist as an “enterotype gradient” of sorts, where multidimensional inequities may be reflected in distinct gut microbial communities’ relative abundance. In fact, some evidence suggests lower SES is associated with reduced gut microbial alpha diversity (66), a phenomenon usually considered detrimental to health (67), and family SES can predict microbial beta diversity in the gut from an early age (68).

The available evidence cannot yet establish a conclusive causal relation between green space access, dietary inequities, NCDs prevalence, socioeconomic background, and the microbiome. Yet, the existing data are arguably sufficient to support the need to enhance opportunities for marginalized populations to protect their health through interventions that restore their microbiomes. Interventions targeting the gut microbiome could diminish health inequities and be part of—or complementary to—broader policy development. An increasing volume of research is suggesting that *biodiverse urban greenspaces* (BUGS) improve urban dweller’s microbiomes with positive consequences for health (69–71). Or as the microbiome rewilding hypothesis purports, “restoring biodiverse habitats in urban green spaces can rewild the environmental microbiome to a state that enhances primary prevention of human disease” (72).

A study that manipulated the plant and microbial diversity of green spaces in a children’s day care center, found that this had an impact on children’s gut and skin microbiomes and enhanced immunoregulatory pathways (52). Another study showed that the biodiversity of the schoolyard environment influences recovery periods of human skin microbiota after its disturbance in children (73). Considering that vegetation cover in Chilean schools is profoundly unequal across the socioeconomic spectrum, with wealthier private schools having much more green cover than their poorer public counterparts (74), there is a great opportunity in Chile to replicate these interventions and apply them to the school environment. This has the potential to diminish health inequities from childhood, by increasing children’s exposure to environmental microbial diversity and providing more recreational spaces for physical activity and social interaction.

Beyond the school environment, considering how unequal urbanization attributes are in Chile, there is an important occasion for public policy to promote healthier cities. The use of targeted *nature-based health interventions/prescriptions*, which aim to expose individuals to somewhat structured nature-based experiences to promote health and wellbeing (75), could enhance exposure to environmental microbes and ascertain beneficial microbial communities within the human microbiota (76). This has the potential to lower the risk of NCDs and their health consequences (77).

When examining the relationships between social inequities, microbial ecology, and health, one can go a step further and consider inequity and the microbiome as enhancers or mediators of a *syndemic*; the complex aggregation of apparently independent diseases embedded in an unequal societal context, exacerbating the adverse effects of each condition (78). Each health condition is exacerbated by socioeconomic disparities, not as simple comorbidities. Approaching the problem from the perspective of a syndemic could aid in the understanding and management of the risk factors promoting the trends previously described, and novel strategies involving microbial rewilding strategies may play a role in this. Similarly, theoretical approaches that *do justice* to such complexity are needed (79–81). Novel, integrative approaches are of utmost importance for the >10% of the Chilean population living below the poverty line and the poorest billion people on the planet (82).

## FUTURE DIRECTIONS

At the time of this writing, Chile is in the middle of a crucial process regarding social equity. A democratically elected constitutional convention is designing a new constitution for the country. This could be the perfect opportunity for policy-makers, politicians, health professionals, environmental health experts, and academics, to collaborate across disciplines and address inequity from a perspective that goes beyond direct economic considerations. Increasing access and availability of urban recreational grounds may not be enough, and perhaps more *biodiverse urban green space* is needed. A potential first step toward the latter is granting constitutional protection to biodiversity (including microbial biodiversity), as the president of the Chilean Society of Microbiology recently suggested (https://somich.cl/la-diversidad-de-los-microorganismos-a-la-constitucion/).

Conversely, educating the population on healthy food choices is an underserved endeavor, and policies regulating the publicity of processed foods should be strengthened. Mental health and NCDs must be addressed in an integrative way; future research should investigate the possibility of an “enterotype gradient” and develop targeted interventions to improve key microbial functions in the gut. Dietary interventions that promote the intake of more fiber have shown that the gut microbiome can quickly change its composition (83, 84).

Furthermore, promoting low-cost, population-based public health interventions encompassing transdisciplinary perspectives can be more cost-effective and successful in the long-term, than spending most budget, time, and effort on mitigating proximal or immediate causes of disease. Addressing inequity implies directing most of our focus on its causes rather than its symptoms; transdisciplinary efforts that integrate microbial ecology, lifestyles, psychology, and brain and medical sciences, may have something important to say in this endeavor (85).
